# Public perception of health technologies: an exploratory spatial mapping of risks, benefits, and value attributions

**DOI:** 10.3389/fdgth.2025.1715810

**Published:** 2026-01-15

**Authors:** Philipp Brauner, Julia Offermann, Martina Ziefle

**Affiliations:** Communication Science, RWTH Aachen University, Aachen, Germany

**Keywords:** health beliefs, health communication, health technologies, medical sociology, risk perception, social acceptance, technology acceptance, trust in physicians

## Abstract

**Purpose:**

The social acceptance of health technologies is crucial for the effectiveness and sustainability of healthcare systems amid the demographic change. However, patients’ acceptance, which shapes technology use and compliance, is still insufficiently understood.

**Methods:**

In this study, we explore how perceived risks and perceived benefits relate to attributed value as a proxy for social acceptance. Unlike most studies that focus on individual technologies, we measure public perception of 20 very different types of health technologies—ranging from plaster cast and x-Ray to insulin pumps, bionic limbs, and mRNA vaccines. Through an online survey utilizing a convenience sample of 193 participants from Germany and Bulgaria, we assessed perceived risks, benefits, and overall value attributed to these technologies. The study presents a visual mapping of the technologies and investigates the individual and technology-related factors shaping these perceptions.

**Results:**

The findings suggest that perceived benefit is the strongest predictor for overall value (*β* = +0.886), while perceived risk plays a significant, but much smaller role (*β* = −0.133). Together, both factors explain 95% of the variance in overall attributed value (95%, *R***^2^** = .959). Further, individual differences, such as prior care experience and trust in physicians, significantly influences the perceptions of health technologies.

**Conclusion:**

We conclude with recommendations for effectively communicating the benefits and risks of health technologies to the public, mitigating biases, and enhancing social acceptance and integration into healthcare systems.

## 1 Introduction

The continued development of healthcare technologies is crucial to the viability of the healthcare sector in an aging society that leads to an increase in age-related diseases and associated health problems (1–4). Diseases, such as cardiovascular disease, diabetes, and cancer continue to rise, and new and evolving infectious diseases, as the recent COVID-19 pandemic (5), threaten the healthcare systems around the world (1, 6).

To face this challenge, the development and implementation of health technologies is crucial to improving health care and the quality of life of people. Health technologies have many advantages (7): They enable better diagnoses (8, 9), more effective treatments, and improved health monitoring (10, 11) and support personalized medicine (12). Given their potential, it is essential that health technologies continue to evolve to meet the continuously changing needs of the healthcare system and promote health and well-being of individuals and the society (13, 14).

Yet, the successful deployment of these innovations depends on more than just technical capability. Thus, the assessment of health technology requires the integration of many perspectives, such as the medical applicability, economic viability, and legal compliance. However, integrating social acceptance is equally crucial in health technology assessment because widespread adoption and effective implementation depend on public trust, cultural alignment, and societal readiness. The patient perspective addresses issues such as accessibility, ease of use, and confidence in the technology (15). It is essential that patients feel comfortable and confident using new health devices or applications, as this can influence their willingness to actively participate in their own healthcare. In addition, insights from the patient and the health professionals' perspective can help break down barriers and facilitate the integration of new technologies into the healthcare system (16, 17). Considering the patient's perspective, how patients feel about these technologies, as well as the role of individual differences is therefore an important step towards the successful and viable implementation of health technologies in the healthcare system.

Previous research in this field has predominantly focused on investigating the acceptance and evaluation of single technologies [see, for example, (18): meta-study on many individual technologies] revealing that the perception of technology-specific benefits, barriers, and risks are relevant factors for the acceptance of health and assistive technologies. Hence, there are many different studies on the perception of health technology and the risk-benefit tradeoffs focusing on individual technologies (18, 19). However, we are not aware of any study that assesses the perception of various different health technologies in a joint study to facilitate their direct comparison.

While traditional frameworks, such as Davis's Technology Acceptance Model (TAM) (20) are robust in predicting the acceptance of interactive Health Information Technologies (e.g., telemedicine apps, wearables), they are less suitable in healthcare contexts where technology use is not under the direct volitional control of the individual, such as many mandatory or non-user-operated, more complex medical technologies (21, 22), e.g., diagnostic imaging or vaccination programs. In these contexts, the patient acts as a recipient of care rather than an active operator of a system, rendering core constructs like Perceived Ease of Use and Intention to Use theoretically ambiguous. Because these decisions depend more on risk-benefit trade-offs than usability, this study adopts the Value-Based Adoption Model (23), positioning perceived value as the primary driver of acceptance.

To provide a comprehensive comparison of 20 diverse health technologies within a unified framework, this study utilizes the micro-scenario methodology (24). This approach enables the simultaneous evaluation, comparison, and visual mapping of various technologies, alongside the reflexive measurement of individual latent personality constructs regarding technology perception. By visually mapping these technologies based on perceived risks and benefits, the study shifts the research focus from isolated assessments to a holistic perspective. Results reveal that perceived benefit is the dominant predictor of value, significantly outweighing risk, while social acceptance is primarily shaped by trust in physicians and care experience. Ultimately, this paper contributes a novel methodology and a comparative mapping of health technologies, offering policymakers actionable evidence to enhance social acceptance through benefit-centered communication strategies.

## 2 Method

### 2.1 Empirical approach

Instead of examining individual health technologies in depths, we assessed a set of 20 different technologies in a single comprehensive survey. Hereto, we asked the participants to evaluate many different health technologies presented as micro scenarios with a short description on few outcome variables each (24). This approach offers three complementary perspectives: First, the average scores for attributed risk, benefit, and value across all participants and technologies can be analyzed, providing an overall assessment of the field of health technologies (“grand mean”). Second, for each assessment variable, the average evaluation for each technology across the participants can be interpreted as a health technology assessment. Using this perspective, one can compare the different technologies, identify outliers, and analyze relationships in the technology evaluations. Third, for each assessment variable, the average evaluations of each participant across the technologies can be interpreted as individual difference (user factor), as it is a reflexive measurement of a latent personality construct through repeated measurements. Using this perspective, one can study, for example, the influence of age, gender, or care experience on health technology attributions.

We compiled the final set of 20 health technologies (see Table 1) through multiple workshops with researchers in the field of health technology acceptance. To ensure a balanced and non-biased selection, we deliberately included technologies spanning diverse categories, such as invasive vs. non-invasive and digital vs. analog applications. Furthermore, we prioritized widely known technologies to ensure sufficient lay familiarity for valid evaluations, explicitly excluding highly specialized or niche devices. We ensured that the selection is not systematically biased (to avoid Berkson's paradox (34), which may cause spurious correlations caused by a selective and biased sample of topics). In the survey, the selected health technologies were presented to the participants with their name and a short description (see Table 1 for the short labels and the full descriptions on the public OSF repository).

**Table 1:** Assessment of the different health technologies by the participants in regard to perceived risk, benefit, and overall perceived value.

Health technology	Mean risk (SD)	Mean benefit (SD)	Mean value (SD)
Plaster cast	−69.0% (SD = 40.6%)	75.7% (SD = 36.4%)	73.1% (SD = 37.5%)
x-ray	−19.1% (SD = 54.4%)	70.1% (SD = 41.5%)	60.9% (SD = 45.7%)
Endoscope	−37.3% (SD = 54.6%)	70.9% (SD = 40.5%)	68.3% (SD = 38.3%)
Analog blood pressure monitor	−80.3% (SD = 37.4%)	78.1% (SD = 42.2%)	80.2% (SD = 35.8%)
Respirator	−48.2% (SD = 52.1%)	76.0% (SD = 38.9%)	74.3% (SD = 41.7%)
Magnetic resonance imaging (MRT)	−56.1% (SD = 49.0%)	85.4% (SD = 31.0%)	83.1% (SD = 30.8%)
Cardiac pacemaker	−40.0% (SD = 51.2%)	74.5% (SD = 36.8%)	71.8% (SD = 36.6%)
Health wearables	−59.5% (SD = 48.9%)	54.8% (SD = 52.2%)	56.7% (SD = 49.5%)
Care apps	−58.4% (SD = 49.2%)	62.5% (SD = 44.9%)	64.5% (SD = 44.0%)
Preventive apps	−48.1% (SD = 51.4%)	42.0% (SD = 54.9%)	46.2% (SD = 49.9%)
Nursing robots	−23.0% (SD = 59.9%)	35.5% (SD = 56.7%)	27.8% (SD = 61.3%)
Robotic surgery	−17.5% (SD = 61.6%)	52.4% (SD = 49.9%)	47.6% (SD = 54.3%)
Electronic patient file	−48.9% (SD = 57.9%)	71.8% (SD = 49.2%)	65.2% (SD = 48.7%)
Home emergency call button	−77.2% (SD = 39.6%)	82.8% (SD = 36.7%)	81.9% (SD = 37.9%)
Insulin pump	−37.4% (SD = 54.1%)	64.1% (SD = 47.2%)	65.8% (SD = 45.5%)
Medical camera monitoring	−51.0% (SD = 53.7%)	64.7% (SD = 47.1%)	57.5% (SD = 51.3%)
mRNA vaccines	5.3% (SD = 67.0%)	21.8% (SD = 71.4%)	18.2% (SD = 70.9%)
Bionic limb	−51.4% (SD = 50.0%)	74.4% (SD = 41.7%)	72.0% (SD = 41.8%)
Syringe	−50.7% (SD = 47.4%)	74.6% (SD = 36.5%)	65.6% (SD = 44.5%)
Heart-lung machine	−22.3% (SD = 62.8%)	69.9% (SD = 42.8%)	67.1% (SD = 43.2%)
Overall Mean (SD)	**−44.5%** **(SD** **=** **52.1%)**	**65.1%** **(SD** **=** **44.9%)**	62.4% (SD = 45.5%)

As dependent variables for the health technology assessment we asked for the perceived risk and perceived benefit of the respective technology, as well as the participant's overall perceived value or attitude towards it (23). Participants evaluated each variable on a single 6-point semantic differential (25, 26) (Risk: *risky–harmless*, Benefit: *useless–useful*, Value: *negative–positive*), making use of their reliability besides their shortness (27), as well as their metric properties and intuitive center of the scale (28).

As explanatory user factors, we asked for the participants demographics, health, and care experience. For the demographics, we asked for the participant' gender (male, female, diverse, no answer), age in years, and the country the participants identify with most. For health- and care-related information (answer options: *yes/no*), we asked the participants if they suffer from a chronic illness. Participants could voluntarily disclose the type of illness they are experiencing. In addition, they reported if they depend on assistance and care in their everyday life. For quantifying private care experience, the participants indicated whether they have already cared for a person in need of care close to them. For the assessment of professional care experience, the participants answered the statement “*My job involves caring for people”*.

For measuring experience with health technology, we selected the four common electrical consumer devices (blood pressure monitor, insulin pump, smartwatch, health apps).

**Figure 1:**
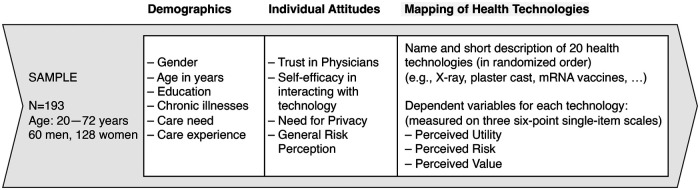
Illustration of the survey with demographics, individual attitudes and the 20 micro-scenarios.

Lastly, we measured different individual attitudes of the participants on 6-point Likert scales using each 4–5 items, i.e., trust in physicians, self-efficacy in interacting with technology, need for privacy, and the participants' general risk perception. Figure 1 illustrates the design of the survey. In total, the survey had 60 items for the health technology mapping and additional 31 items for capturing participants' demographics, health and care experience, and their individual attitudes.

### 2.2 Data acquisition, filtering, coding, and analysis

We used an online survey in Qualtrics to collect responses. To mitigate bias, we randomized the order of the presented health technologies. For test efficiency, we kept the order of the three target items (24). Utilizing convenience sampling, we distributed the survey via personal (e.g., personal email) and social networks (e.g., Facebook, LinkedIn). Due to the researchers' social networks, the questionnaire was available in English, Bulgarian, and German.

At the beginning, participants provided informed consent and after being informed that participation was voluntary, uncompensated, anonymous, and that they could quit the survey at any point without any negative consequences. Participants below the legal age of 18 could not take part in the survey. In accordance with the regulations of the German Psychological Society (29) and the requirements of our funding agency, ethical approval by an institutional review board was not required due to the nature of the survey and the informed consent procedure.

To facilitate interpretation and visual mapping, the study's core variables (perceived risk, perceived benefit, and attributed value) were recoded to a scale ranging from −100% to +100%. In this system, 0% represents a neutral evaluation (the origin of the coordinate system), while the endpoints reflect the semantic extremes (e.g., −100% for maximum safety/uselessness and +100% for maximum risk/usefulness).

Depending on the measurement quality, we analyzed the data with non-parametrical and parametrical methods (Pearson's r and Kendell's *τ* correlations, OLS-multiple linear regressions). Following the conventions in the social sciences, we set the level of statistical significance to *α* = .05. We only included participants from Bulgaria and Germany, as all other countries had less than 10 participants. Further, we cleaned the sample from speeders (participants faster than 40% of the median survey duration of 14.4 min.) as this criterion is usually sufficient for identifying meaningless responses in online surveys (30). We used R version 4.5.1 for the analyses. All research materials, data, and analysis scripts are publicly available on the website of the Open Science Foundation. https://osf.io/shvg7/. Based on the reviewer's suggestion, a *post-hoc* power analysis (G*Power) was calculated to assess the robustness of the regression analysis. With an assumed effect size of *f*^2^ = .25 and a power of.95 for two predictors, the required sample size was 65. Our sample of 193 participants is therefore sufficient for the primary model, although too limited for detailed cross-cultural comparisons.

### 2.3 Description of the sample

After cleaning the sample consisted of 193 participants in the age ranged between 20 and 72 years with a median age of 44 years (SD = 13.6). 31.1% of the participants (*n* = 60) identified themselves as male, 68.4% as female (*n* = 132), and one participant did not answer this question (0.5%). *N* = 142 (71.5%) participants were from Bulgaria and 51 (26.4%) participants came from Germany. Gender and age were not correlated in this sample (τ = .033, *p* = .535), however country was associated with both age (*τ* = .384, *p* ≤ .001) and gender (*τ* = .159. *p* = .028), with the sample from Bulgaria being older and having more female participants (see Limitations). The sample was fairly educated with as the majority of 75.6% (*n* = 146) indicated to have a university degree and 3.1% (*n* = 6) a doctoral degree, while only 21.2% (*n* = 41) reported lower educational degrees (e.g., university entrance degree, secondary school certificates).

Asked for health characteristics, a quarter of the participants (25.4%, *n* = 49) reported suffering from a chronic illness, and mentioned examples referred to typical age-related illnesses, such as diabetes and high blood pressure. In line with this, a minority of the sample indicated to depend on care (12.2%, *n* = 23). Beyond that, the participants were asked for their experiences in healthcare. Here, a proportion of 43.1% (*n* = 81) reported to have already cared for a person in need of care close to them. Asked for professional experience, a minority of 12.8% (*n* = 24) confirmed that their job deals with caring for people.

## 3 Results

### 3.1 Grand mean of health technology perceptions

**Figure 2:**
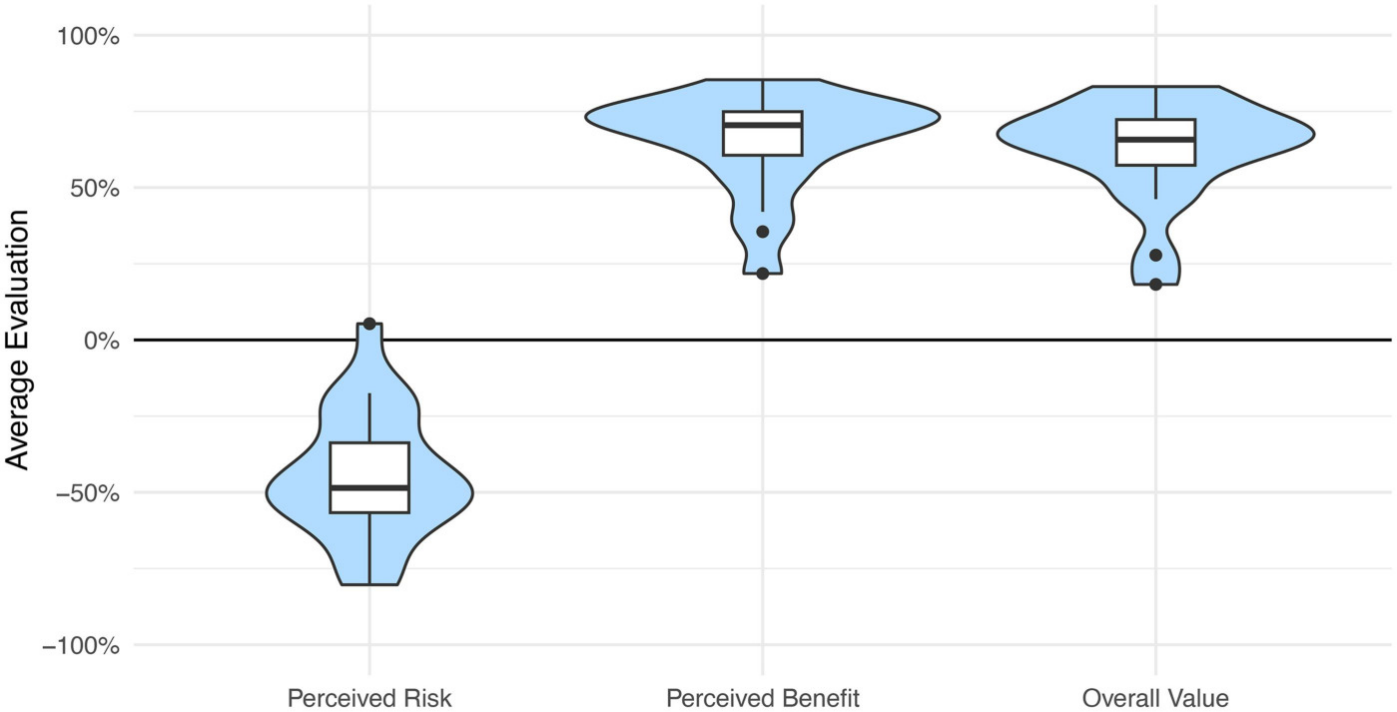
Illustration of the assessment of the 20 different health technologies in terms of perceived risk, benefit, and value by the 193 participants. On average, participants from both countries see heath technologies as safe, useful, and positive. The boxplots shows median, 25th/75th percentile and the background shape shows the frequency distribution of the evaluations for each dimension.

The average perceived risk across all participants and all technologies is −44.4% (“SD” = 56.4%), the average benefit is +65.0% (“SD” = 48.4%) and the average perceived overall value of +62.4% (“SD” = 49.0%). Consequently, on average, the set of health technologies in the study is perceived as rather safe and useful, and the participants have a positive overall attitude towards these. Figure 2 visualizes these findings.

In the next sections, we analyze the results first in terms of health technology attributions (how the participants evaluated the different technologies in this study) and second as individual differences (interpreting the averaged attributions for each participant as measurements of latent personality constructs).

### 3.2 Technology perspective: perception of individual health technologies

In this section, we analyze the evaluation of individual health technologies, averaged across all participants. The health technologies perceived as least risky were the analog blood pressure monitor (−80.3%), the home emergency call button (−77.2%), and the plaster cast (−69.0%). The technologies with the highest perceived risk were the x-ray (−19.1%), robotic surgery (−17.5%), and mRNA vaccines (+5.3%). As the positive risk score indicates, only the last was perceived as slightly risky.

On perceived benefit, the analog blood pressure monitor (+78.1%), the home emergency button (+82.8%), and magnetic resonance imaging (MRT) (+85.4%) were rated as most useful. Whereas preventive healthcare apps (+42.0%), nursing robots (+35.5%), and mRNA vaccines (+23.8%) were perceived as less useful, although still on the positive side of the scale.

Regarding the perceived value a similar pattern emerged: The analog blood pressure monitor (+80.2), the home emergence button (+81.9%), and magnetic resonance imaging (+83.1%) were evaluated as the most positive health technologies, whereas preventive healthcare apps (+46.2%), nursing robots (+27.8%), and mRNA vaccines (+18.2%) received the lowest, but still positive evaluations. Table 1 lists the assessment of every technology.

**Figure 3:**
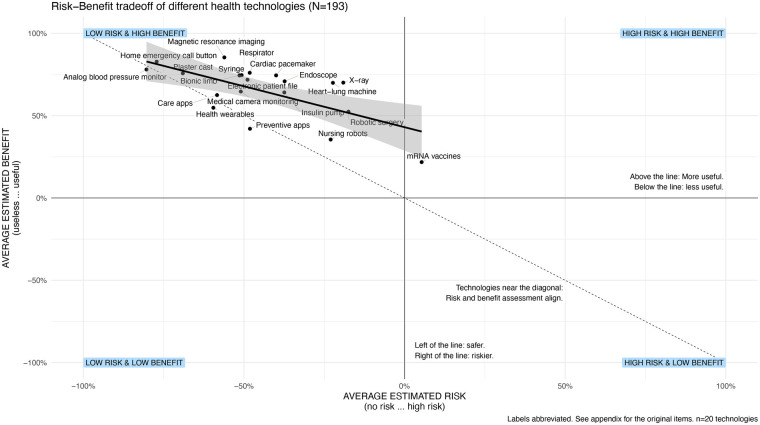
Illustration of perceptions of different health technologies in terms of risk and benefit and the resulting regression line. Most health technologies are seen as rather useful and rather safe and risk is associated with benefit. The thin grey diagonal illustrates a balance between perceived risk and perceived benefits.

Next, we interpret the perceived risk and benefit scores of each technology as coordinates and place the technologies on a spatial map that can be interpreted in three ways (Brauner 2024): First, the distribution of the topics in regard to the studied dependent variables can be analyzed, e.g., how breadth or narrow is the distribution of technologies. Second, the map can be analyzed for outliers. Third, the bivariate or multivariate relationships can be interpreted, e.g., how strong is the relationship between risk and benefit. Figure 3 shows that most technologies lay in the upper left quadrant of the map with technologies with higher benefit and lower risk. Only mRNA vaccines are in the quadrant of the higher risk and higher benefit health technologies.

Overall, the assessment dimensions perceived risk, benefit and overall value are strongly correlated. There is a significant and strong negative correlation between Risk and Benefit (*r* = −.647, *p* = .002) and risk and overall value (*r* = −.729, *p* = .001). The significant correlation between benefit and overall value is positive and strong (*r* = .973, *p* < .001).

To disentangle the mutual correlations, we calculated a multiple linear regression with risk and benefit as independent and overall value as dependent variable. The results of the regression yield a significant model with the two predictors risk and benefit explaining 96.5% of the variance of overall value ($R_{adj}^{2}=.959,\;F(2,17)=224.2,\;p<.001$). The influence of risk on overall value ($\beta_{\mathrm{Risk}}=-0.133,\;p<.001$) is much smaller than the influence of usefulness ($\beta_{\mathrm{Benefit}}=0.886,\;p<.001$). Following expectations, higher risk has a negative influence of overall value, while higher benefits have a positive influence. Table 2 details the regression model.

**Table 2:** Significant model of the multiple linear regression with overall value as dependent variable and the average risk and benefit scores of the 20 health technologies as independent variables ($R_{adj}^{2}=.959$).

Predictor	*B*	SE	*β*	*T*	*p*
(Intercept)	0.012	0.032	—	−0.381	.708
Risk	−0.134	0.048	0.133	−2.789	.013*
Benefit	+0.886	0.062	0.886	14.190	<.001***

*p < .05, **p < .01, ***p < .001.

### 3.3 Individual perspective: person-specific factors in risk, benefit and value perceptions

In this section, participants' evaluations are interpreted as individual differences, with their responses considered repeated reflexive measurements of latent personality constructs (24). First, we analyzed if individual characteristics of the participants (demographics and attitudes) influence the evaluation of health technology, i.e., the perception of risk, benefit, and value across all investigated technologies (see Table 3). The results revealed that the demographic characteristics of the participants—age, gender, and educational level—did not correlate with the evaluations of risk, benefit, and value (n.s.). Focusing on health- and care-related characteristics, the participants' health status was not significantly associated with the evaluation of health technology (n.s.). In contrast, the care-related characteristics care need, i.e., “*being dependent on care*” (benefit: *r* = .166, *p* < .05; value: *r* = .188, *p* < .05), and private care experience, i.e., “having already cared for family member in need of care” benefit: *r* = .166, *p* < .05; value: *r* = .211, *p* < .05), were both weakly related with the evaluations of benefit and value. In contrast, professional care experience did not have any effect on the evaluation of health technologies (n.s.). From the attitudes investigated, neither the self-efficacy in interacting with technology (n.s.) nor the participants' need for privacy (n.s.) was significantly correlated with the evaluation of health technology. In line with this, also the participants' general risk perception and behavior (n.s.) did not reveal significant relationships with the evaluations of risk, benefit, and value. In contrast, trust in physicians based on previous experiences represented the own attitudinal variable which was significantly correlated with all three dimensions of evaluating health technology: perceived risk (*r* = −.186, *p* < .05), perceived benefit (*r* = .193, *p* < .01), and overall value (*r* = .194, *p* < .01).

**Table 3:** Correlations between user diversity factors and the individuals’ perception of health technologies in terms of risk, benefit, and attributed value.

Predictor	Risk	Benefit	Value
Age	−.030	−.062	.031
Gender	.130	-.008	−.030
Education	.039	.078	.092
Health status	−.024	.084	.001
Care need	−.101	.166*	.188*
Private care experience	−.138	.166*	.211**
Prof. care experience	.014	.082	.080
Self-efficacy technology	−.094	.116	.092
Need for privacy	.110	−.060	−.064
General risk perception	−.023	−.041	.044
Trust in physicians	−.186*	.193**	.194**

*p < .05, **p < .01, ***p < .001.

To investigate the influence of the three significant user diversity factors on the participants' evaluation of the value of health technology, we calculated a multiple-linear regression with overall perceived value as dependent and care need, private care experience as well as trust in physicians as independent variables. Table 4 presents the significant model. Overall, the three independent variables explained 9.9% of the variance of the overall value ($R^{2}=.099,\;F(3,174)=6.34,\;p<.001)$ and are significant predictors of the overall value.

**Table 4:** Significant model of the linear regression with overall value as dependent variable and the user diversity factors (care need, private care experience, and trust in physicians) as independent variables.

Predictor	*B*	SE	*β*	*T*	*p*
Care need	.133	.064	.480	2.090	.038*
Private care experience	.096	.041	.347	2.339	.020*
Trust in physicians	.050	.021	.170	2.345	.020*

*p < .05, **p < .01, ***p < .001.

## 4 Discussion

In this study, we surveyed participants of diverse ages, genders, and nationalities to evaluate a broad set of health technologies, ranging from established tools to recent innovations. Unlike prior research that often focuses on isolated technologies [see (18) for a systematic review], our aim was not to detail specific health technologies but to capture the breadth of the field through two distinct perspectives: First, we applied a technological perspective, comparing how different technologies are evaluated relative to one another. Second, we analyzed an individual perspective (interpreting technology perceptions as reflexive measurement of an individual difference), examining how general perceptions of risk, benefit, and value relate to user characteristics.

For the first perspective, the results indicate that the set of queried health technologies, on average, are seen as rather safe. The overall perceived value of health technologies is mostly explained by the benefit attributed by the participants and to a much lesser extend by the perceived risk of using it. Beyond prior research that identified the perception of benefits and risks for the acceptance of health assistive technology [see (18) review], our approach enabled the quantification of the relationships between benefit, risk, and acceptance for a broad variety of different health technologies. In line with prior research (31, 32), the present findings demonstrate a negative correlation between perceived risks and perceived benefits, alongside an inverse association with overall value and acceptance evaluations. Notably, these relationships were consistently observed across a diverse set of 20 health technologies, thereby reinforcing the robustness and generalizability of the established patterns.

For the second perspective, this study corroborates the prior findings that care experience influences the evaluation of the benefits and the acceptance of health technologies (33). Results further suggest that trust in physicians represents a key factor in the risk-benefit-perception as well as the acceptance of health technologies.

Our analyses revealed significant relationships between individuals' care needs, previous care experiences, and trust in physicians with the perceived value of health technologies. These findings have direct implications for practical implementation. In terms of clinical communication, the pivotal role of trust in physicians suggests that adoption strategies should not rely solely on technical marketing but should leverage healthcare professionals as trusted ambassadors. Policymakers should therefore prioritize initiatives that empower physicians to introduce digital tools, ensuring that the existing patient-doctor trust transfers to the technology. Regarding technology design, the influence of prior care experience implies that developers must better accommodate users lacking such background; this calls for co-design processes involving informal caregivers to ensure interfaces align with the intuitive mental models of care. By moving beyond broad demographic targeting to address these specific experiential and trust-based factors, healthcare systems can better align innovations with user needs and significantly enhance adoption rates.

Focusing on communication strategies related to health technology, discussions in the media are often framed by the perceived risks. Our study, however, suggests that people rather build on the perceived benefits for forming their overall assessment in terms of the overall value. While transparent communication on both the risks and benefits of health technology is important. Our results suggest that focusing on communicating the benefits is the greater lever for the perceived value and thus the acceptance of health technology than mitigating the perceived risks.

This article analyzed the technology-related risk-benefit tradeoffs and how individual factors, such as care experience, influence perceptions of risks, benefits, and overall value. While the assessment of health technology requires more than just social perspectives, low social acceptance can lead to reduced adoption or increased fear and resistance. Therefore, integrating social acceptance and the findings from this study alongside medical, economic, ethical, and legal considerations is crucial for ensuring the successful implementation and effectiveness of health technology in real-world applications.

## 5 Limitations and future research

This study has limitations. First, the presented study complements research on the detailed evaluation of a single health technologies by analyzing many using micro scenarios. Hence, the assessments are based on an affective evaluation of the technologies and not on a deliberate weighting of the benefits and barriers. Our approach cannot explain the reasons for the evaluations, which would be subject to further studies. Second, the study builds on a convenience sample and thus exhibits demographic biases, including a high prevalence of women and university-educated participants, alongside a cross-country disparity where the larger Bulgarian cohort was significantly older and more female-dominated than the German group. However, as our analysis found no significant correlations between these demographic factors and the technology evaluations, the core findings regarding risk-benefit trade-offs appear robust despite these limitations. Hence, the results can inform researchers and policymakers about both, the risk-benefit trade-offs, as well as the relative positioning of health technology. Further, the study can serve as a basis for a population-representative evaluation of various health technologies across Europe and its different healthcare systems. But of course, future work should extend this study by utilizing larger and more representative samples. Finally, the set of technologies examined in this study was intentionally broad and heterogeneous, spanning diverse application contexts and levels of user interaction. This heterogeneity reflects our aim to capture overarching perception patterns across a wide spectrum of health-related innovations rather than to derive technology-specific classifications. Equally, and following the micro scenario's argument that people can have diverging views on specific technologies (18), we did not calculate reliability metrics. Nevertheless, such variation may conceal underlying perception clusters that could emerge when grouping technologies with similar characteristics. While exploring these potential groupings lies beyond the scope of the present study, future research would benefit from applying clustering approaches to identify perception-based categories of health technologies and to examine whether such clusters provide additional explanatory value.

## 6 Conclusion

This study charted the public perception of 20 diverse health technologies to provide a comparative framework for understanding social acceptance. Our findings demonstrate that while perceived risk is a significant factor, perceived benefit is the dominant predictor of the overall value attributed to health technologies. Furthermore, acceptance is not merely a technical calculation but is deeply influenced by individual human factors, specifically trust in physicians and prior care experience. Based on these insights, we derive the following recommendations:

*For Policymakers and Health Communicators:* Strategies to foster technology adoption should shift focus from solely mitigating risk perceptions to actively highlighting tangible benefits. Since perceived benefit explains the vast majority of variance in acceptance, communication campaigns must clearly articulate how innovations improve individual health outcomes and quality of life. Transparency regarding risks remains necessary, but it should not overshadow the utility of the technology.

*For Researchers:* This study validates the utility of using micro-scenarios for comparative technology mapping. We recommend that future research moves beyond the evaluation of isolated technologies to adopt unified frameworks. This comparative approach allows for the identification of relative outliers and broader patterns in public perception that single-case studies may miss.

*For Health Practitioners and the Healthcare Community:* The strong link between trust in physicians and technology value suggests that medical professionals are the critical bridge for digital adoption. Successful implementation requires that physicians are not only equipped to use these technologies but are also supported in maintaining strong, trust-based relationships with their patients.

Ultimately, the successful integration of health technologies into society depends on aligning technical innovation with human needs. By prioritizing benefit-oriented communication and leveraging the trusted physician-patient relationship, healthcare systems can better ensure that technological advancements translate into accepted and effective care solutions.

## Data Availability

The datasets presented in this study can be found in online repositories. The repository can be found below: https://osf.io/shvg7/.
